# Global burden of skin and subcutaneous disease across childhood and adolescence in 204 countries and territories, 1990-2021

**DOI:** 10.1016/j.jdin.2026.05.015

**Published:** 2026-05-25

**Authors:** Jiehua Wei, Zhiwei Wang, Yuxuan Chen, Yi Wang, Xiaochen Dai, Joel M. Gelfand, Hervé Bachelez, Yi Xiao, Minxue Shen, Xiang Chen

**Affiliations:** a Department of Social Medicine and Health Management, Xiangya School of Public Health, Central South University, Changsha, China; b Institute for Health Metrics and Evaluation, University of Washington, Seattle, Washington; c Department of Health Metrics Sciences, School of Medicine, University of Washington, Seattle, Washington; d Department of Dermatology, University of Pennsylvania, Philadelphia, Pennsylvania; e Department of Biostatistics, Epidemiology and Informatics, Perelman School of Medicine, University of Pennsylvania, Philadelphia, Pennsylvania; f Department of Dermatology, Hôpital Saint-Louis, APHP, Paris, France; g Laboratory of Genetics of Skin Diseases, Imagine Institute for Human Genetic Diseases, Université Paris Cité, Paris, France; h Department of Dermatology, Xiangya Hospital, Central South University, Changsha, China; i Division of Public Health and Cohort Study, Furong Laboratory, Changsha, China; j Hunan Key Laboratory of Skin Cancer and Psoriasis, Changsha, China; k Division of Geriatric Skin and Immune Disorders, National Clinical Research Center for Geriatric Disorders (Xiangya Hospital), Changsha, China

**Keywords:** childhood and adolescence, disease burden, epidemiology, health inequality, skin and subcutaneous disease

*To the Editor:* Skin and subcutaneous diseases are a leading cause of nonfatal disease burden globally, yet their impact on children and adolescents warrants special attention: skin diseases during this developmental period impair psychosocial functioning and mental health, while the disease spectrum—from infectious conditions prevalent in low-resource settings to immune-mediated diseases common in high-income regions—differs from that in adults. Using the Global Burden of Disease Study 2021, which integrates population-based surveys, hospital records, and disease registries across 204 countries from 1990 to 2021, we quantified the burden and inequalities of skin diseases in individuals aged 5-19 years, focusing on incidence, prevalence, and years lived with disability (YLDs) across 3 age subgroups: 5-9, 10-14, and 15-19 years.^1^

In 2021, an estimated 1.09 billion incident cases, 550.2 million prevalent cases, and 13.0 million YLDs from skin and subcutaneous diseases occurred in children and adolescents globally (Fig 1). Skin diseases contributed to 11.64% of all YLDs in this population and ranked as the second leading cause of nonfatal disease burden worldwide—having risen from third place in 1990. The global age-standardized incidence rate, prevalence rate, and YLDs rate were 55,136.5, 27,755.1, and 658.2 per 100,000 persons, respectively, with estimated annual percentage changes of 0.34 (95% CI: 0.32-0.37), 0.24 (95% CI: 0.22-0.25), and 0.11 (95% CI: 0.09-0.12), indicating consistent upward trends over the study period.

**Fig 1:**
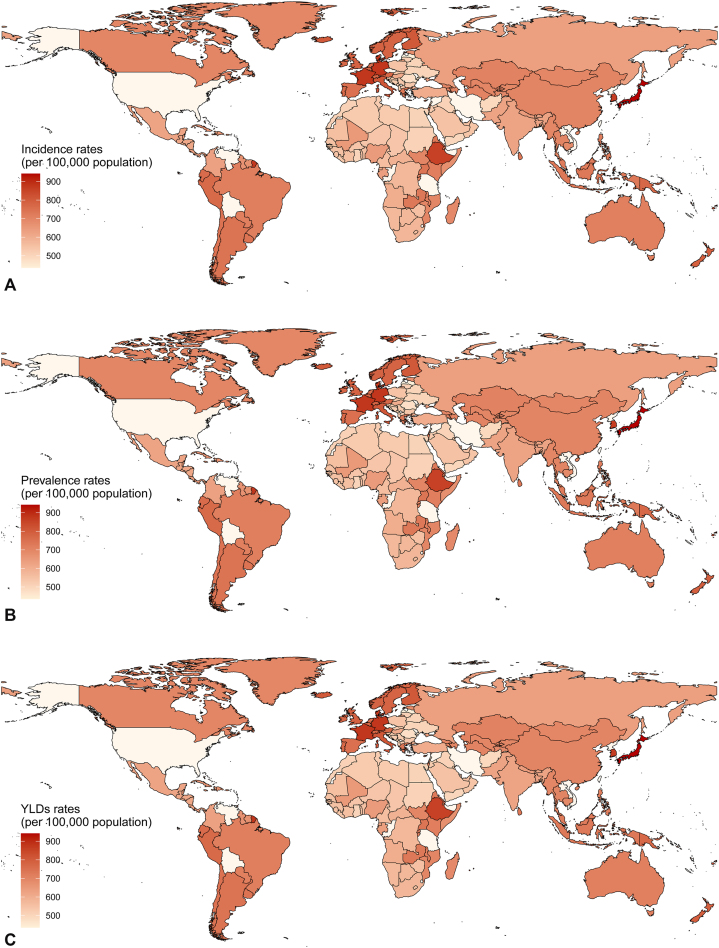
Global age-standardized incidence, prevalence, and YLDs rates for skin and subcutaneous diseases among individuals aged 5-19 years across 204 countries and territories, 2021. **A,** Age-standardized incidence rate; **(B)** Age-standardized prevalence rate; **(C)** Age-standardized YLDs rate. Rates are per 100,000 population. *YLDs*, Years lived with disability.

Disease burden varied substantially by socioeconomic level. High-SDI countries had the highest age-standardized YLDs rate (766.3 per 100,000), whereas low-SDI countries had the highest age-standardized incidence rate (74,712.4 per 100,000) and age-standardized prevalence rate (33,061 per 100,000). This pattern likely reflects a combination of greater infectious skin disease burden in resource-limited settings and more comprehensive diagnosis, surveillance, and documentation of chronic conditions in wealthier countries. Concentration curve analysis confirmed that bacterial and fungal skin diseases were disproportionately concentrated in lower-SDI populations, while psoriasis and alopecia areata were more prevalent in higher-SDI regions.^2^

Sex-based differences were also apparent. Females had consistently higher age-standardized YLDs rate than males across all SDI levels (698.5 vs 620.3 per 100,000 globally), likely reflecting hormonal influences, behavioral factors such as hair styling practices (eg, scalp psoriasis), and greater psychosocial burden associated with visible skin diseases.^3^ Age-specific patterns differed by condition: dermatitis accounted for the highest YLDs in children aged 5-9 years, while acne dominated in adolescents aged 10-19 years (Fig 2). Among the top 20 causes of YLDs across all conditions, skin diseases featured prominently in all age subgroups.

**Fig 2:**
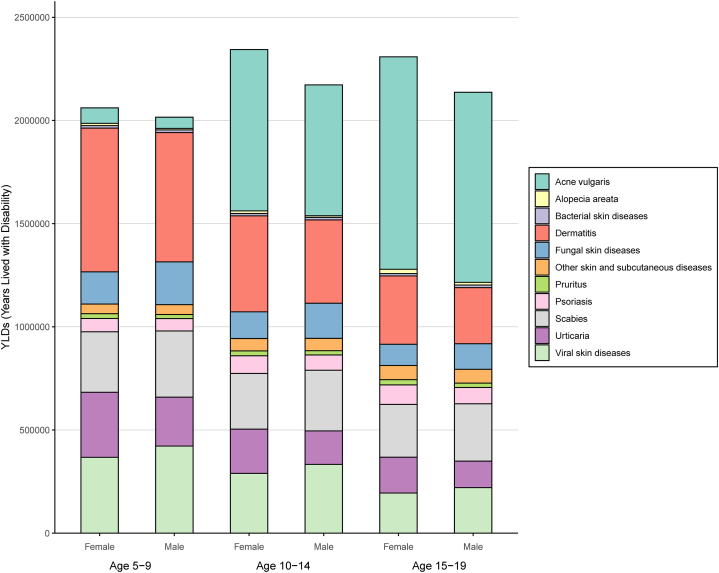
YLDs from skin and subcutaneous disease, age 5 to 19 years. Number of YLDs is age specific rather than cumulative, that is, YLDs from the older age groups do not incorporate the YLDs recorded for the younger age groups. *YLDs*, Years lived with disability.

This study is subject to limitations inherent to GBD methodology, including reliance on modeled estimates in data-sparse regions and the use of uniform disability weights across age groups, which may not fully reflect children's and adolescents' perspectives. Our analysis covers ages 5-19 years per GBD analytical categories; future studies should examine the 0-4 year group to capture the full pediatric spectrum.^4^^,^^5^ In conclusion, skin diseases represent a significant and growing nonfatal burden in children and adolescents globally, with persistent inequalities by sex and socioeconomic level. Region-specific prevention strategies and improved access to dermatological and psychosocial care are urgently needed.

## Conflicts of interest

None disclosed.
